# Recycled Rubber as an Aggregate Replacement in Self-Compacting Concrete—Literature Overview

**DOI:** 10.3390/ma11091729

**Published:** 2018-09-14

**Authors:** Robert Bušić, Ivana Miličević, Tanja Kalman Šipoš, Kristina Strukar

**Affiliations:** Faculty of Civil Engineering, University of Osijek, Vladimira Preloga 3, Osijek HR-31000, Croatia; rbusic@gfos.hr (R.B.); tkalman@gfos.hr (T.K.Š.); kstrukar@gfos.hr (K.S.)

**Keywords:** self-compacting concrete, rubberized concrete, recycled rubber, aggregate replacement

## Abstract

In the past few decades, due to the exponential increase of the world’s population, the number of discarded waste tires has become a serious ecological and environmental problem. Decomposition of waste tire rubber can take longer than 50 years, and every year the number of discarded tires is rapidly growing. With the inclusion of waste tire rubber into self-compacting concrete this global problem can be reduced. Waste tire rubber can be incorporated in self-compacting concrete by partially replacing the natural fine and coarse aggregate, reducing consumption of sand and gravel and preserving these natural materials. In addition, recycling and reusing waste tire rubber avoids the need for tire landfilling, as one of the major ecological problem of the near future. Replacement of natural aggregate with waste tire rubber can have an undesirable influence on the mechanical properties of self-compacting concrete, i.e., compressive strength, flexural strength, splitting tensile strength, and modulus of elasticity, however. On the other hand, replacing natural gravel or sand with waste tire rubber can improve impact resistance, ductility, and fatigue resistance. This paper presents an overview of the literature investigating recycled waste tire rubber used as a fine and/or coarse aggregate replacement in self-compacting concrete and its influence on several essential fresh and hardened self-compacting concrete properties.

## 1. Introduction

The number of unusable waste tires from different kinds of vehicles is rapidly growing and is in fact turning out as one of the major ecological and environmental problems of the present day. Nearly one-billion waste tires are discarded each year [1], and are predicted to be almost 1.2 billion per year by 2030 [2]. Nearly 8.3 million tons of waste tires are discarded per year just in Europe and United States of America (USA) [3], some of which were probably disposed illegally, compromising human health as the aesthetics of nature. In the US, 289 million of waste tires are disposed annually [4]. In Australia, this number is near 51 million [5] and because of the overwhelming number of disposed tires, landfills are being cluttered with scrap tires and causing additional exposure to potential environmental threats, such as mosquitoes, mice, other insects, rats, and an increased risk of fire hazards [4,6]. 

Furthermore, common ways of disposing of the scrap tires, such as landfilling and burning, may cause grave ecological problems, either because of the fast depletion of the site or air pollution, respectively [6,7,8]. According to the European Tyre and Rubber Manufacturers Association (ETRMA), the number of end of life tires (ELT) has grown from 2.48 to 2.88 million tonnes from year 2004 to year 2013 [9]. Still, thanks to different types of associations across the world that promote a circular economy and sustainable development, recycling of waste tires and reuse of tire derived rubber has become very popular in the last few years. Recycling of waste vehicle tires has become very popular topic among scientists and engineers.

Due to the fact that the waste tire rubber, as a non-biodegradable material, has a relatively long lifetime, interest in replacing natural river aggregate in concrete mixtures with rubber derived from waste tires, i.e., rubberized concrete (RC), has attracted the attention of civil engineers and building industry to provide environmental-friendly concrete with recycled tire rubber.

The concrete industry is one of the biggest industries in the world. According to the European Ready Mixed Concrete Organization statistics (ERMCO), the average concrete production in 2015 in the European Union (EU), Russia, USA, and Japan was 344.3, 62.0, 365.0 and 138.0 million m^3^, respectively [10]. Overall, the world produces around 5 billion tonnes of concrete a year [11]. From an ecological point of view, by implementing rubber derived from waste tires in concrete, the amount of disposed waste tires would become smaller and provide a source of eco-friendly concrete. From an engineering point of view, adding waste tire rubber into concrete could produce a material with improved dynamic and durability properties, such as ductility, damping capacity, chloride-ion penetration resistance, carbonation resistance, etc.

Similar to traditional concrete, self-compacting rubberized concrete however has lower levels of emitted radiation and thus safer to building users. Because of its high ductility, improved impact resistance, and energy dissipation properties [12], rubberized concrete has been used in several applications so far, i.e., road barriers, sidewalks, and pavement [13,14,15]. Murugan et al. [16] concluded that the rubberized concrete could be used in sidewalks, sport courts, and traffic noise barriers on highways, i.e., nonstructural applications. Nevertheless, because of its improved impact resistance and ductility, other researchers have suggested that self-compacting rubberized concrete could be used for structural elements subjected to dynamic loads [13]. Before this can be accomplished, it is suggested that experimental investigation on self-compacting rubberized concrete needs to be carried out.

The main objective of this review article is to provide an overview of fresh and hardened properties of self-compacting concrete with partially replaced fine and/or coarse aggregate with waste tire rubber. The fact that fine and coarse aggregate makes approximately 50% of weight of the self-compacting concrete can be used as a motivation to thoroughly study the influence of partially replacing natural aggregate with waste tire rubber, not only to provide lightweight self-compacting concrete (SCC) and to preserve natural resources, but also to maintain or even improve the mechanical properties and durability of SCC.

## 2. Mixing Procedure and Rubber Aggregate Properties

**Figure 1:**
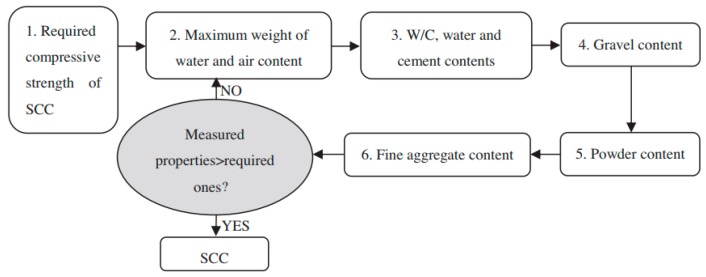
Mixture design procedure of method proposed by Ghazi—compressive strength method [18]. Reproduced with permission from C. Shi, Z. Wu, K. Lv and L. Wu, A review on mixture design methods for self-compacting concrete; published by Elsevier, Construction and Building Materials, 2015.

**Figure 2:**
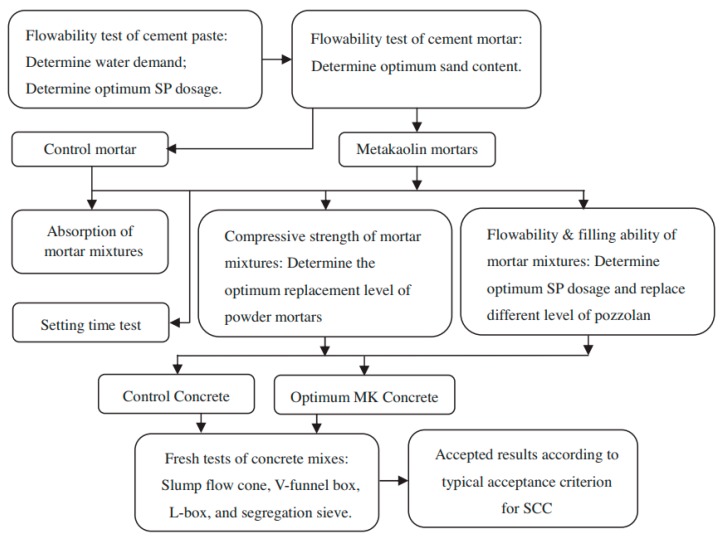
Mixture design procedure of method proposed by Khaleel – empirical design method [18]. Reproduced with permission from C. Shi, Z. Wu, K. Lv and L. Wu, A review on mixture design methods for self-compacting concrete; published by Elsevier, Construction and Building Materials, 2015.

Currently, there is no standardized methodology to design mixes of self-compacting rubberized concrete [17]. However, there are several mixture design methods for self-compacting concrete. Shi et al. [18] classified these methods in five categories: empirical design methods, compressive strength methods, close aggregate packing methods, statistical factorial models, and rheological paste models. Flow charts of two relatively new mixture design procedures published in 2014 and 2013 are given in Figure 1 and Figure 2, respectively.

So far, there is no mixture design method that fully meets all of the usual requirements, i.e., widely applicable, fit a variety of technical requirements, sustainability, strong robustness for variable raw materials, and cost [18]. However, to avoid SCC fresh state problems, i.e., segregation and bleeding, Najim and Hall [17] summarized the mixing procedures of several authors and proposed a few important mixing steps:

- all dry mix components should first be mixed from 1 to 5 min before progressively adding water;
- after water is added, mixing should continue for another 3 to 5 min;
- satisfactory results can be obtained by mixing aggregates and powder for 30 s, without adding any water, then adding one-third of the required superplasticizer, and then mixing for an additional 90 s; and,
- final step is to add the remaining amount of superplasticizer with all other admixtures and mix for an extra 210 s.

**Figure 3:**
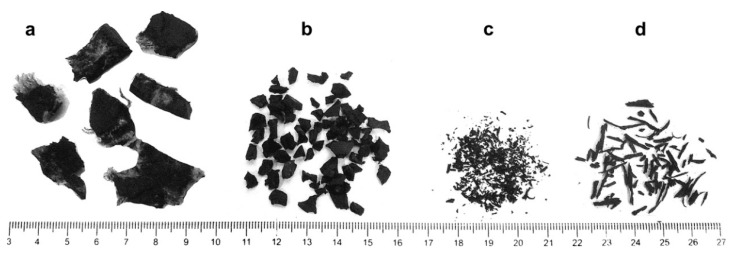
Categorization of waste rubber aggregates: (**a**) chipped, (**b**) crumb, (**c**) granular and (**d**) fibre [17]. Reproduced with permission from K. B. Najim and M. R. Hall, A review of the fresh/hardened properties and applications for plain- (PRC) and self-compacting rubberised concrete (SCRC); published by Elsevier, Construction and Building Materials, 2010.

Pretreatment of rubber aggregate is also desirable to improve adhesion between waste tire rubber and cement matrix. Waste tires have been used in experimental investigations to partially replace the aggregate in traditional concrete and self-compacting concrete for two and a half decades. Mostly, fine and coarse aggregate were replaced with rubber aggregate from mechanical grinding of end-of-life tires. Rubber particles in concrete are obtained from waste tires. There is a mechanical procedure occurring either at cryogenic or ambient temperatures. As can be seen in Figure 3, the waste tire rubber can be classified into four categories, depending on rubber size, rubber shape, and replaced material [17]

- Chipped/shredded rubber aggregate (coarse rubber), used as coarse aggregate replacement, i.e., replacement for natural gravel; rubber particles are between 13 and 76 mm.
- Crumb rubber, commonly used as sand replacement; grain size is between 0.425 and 4.75 mm.
- Ground rubber, used as cement replacement; grain size is less than 0.425 mm.
- Fibre rubber aggregate; the shredded rubber is in the form of short fibres with an average of 12.5 mm length.

## 3. Fresh SCC Properties

### 3.1. Flowability, Viscosity, Passing Ability and Segregation Resistance

Regardless of rubber type, rubber size, rubber replacement level, and replaced material, most authors have reported similar fresh SCC behaviour, i.e., a reduction in flowability, passing ability and segregation resistance, and an increase in viscosity with an increase of rubber content. A few authors have claimed essentially the opposite, i.e., Mishra and Panda [19] reported an increase in flowability and the reduction in viscosity and Ganesan et al. [20], Zaoiai et al. [21], Mishra and Panda [19] reported an increase in passing ability with an increase of rubber aggregate replacement level. Si et al. [14], however, described a reduction in flowability and an increase in viscosity due to increase in rough surface and surface friction of rubber particles. A similar explanation was given by AbdelAleem and Hassan [22], who explained similar fresh SCC behaviour as being caused by the sharp edges of rubber particles causing blockages; more energy is required to move rubber particles because of these sharp edges, hence a reduction in flowability and passing ability occurs.

Replacement levels, as given in Table 1, show that only a few authors replaced natural aggregate with rubber aggregate above level of 40% (Emiroğlu et al. [6], Ismail and Hassan [7], and Uygunoğlu and Topçu [23]); in most experimental investigations the replacement level was 15% (Bideci et al. [24]) or 20% (Ganesan et al. [20], Mishra and Panda [19], Zaoiai et al. [21]), all for the purpose of maintaining acceptable fresh SCC properties. Except for the similar final test results given above, most authors were prone to use different kind of chemical admixtures to maintain or even to improve fresh SCC properties, i.e., superplasticizer (SP) [1,5,6,19,20,23,24,25,26,27]; viscosity modified admixture (VMA) [5,20,26,27]; or, high range water reducer admixture (HRWRA) [7,14,22,28,29].

To improve the bond connections between rubber aggregate and binder, Ganesan et al. [20] pretreated rubber aggregate with poly-vinyl alcohol, Aslani et al. [5] using a water soaking method and Si et al. [14] with sodium hydroxide (NaOH). Si et al. [14] reported reduction in flowability of self-compacting rubberized concrete (SCRC) when the rubber particles were pre-treated with NaOH; the water soaking method proved to be a cost effective method to improve bonding [5].

Most of the test results that are discussed above were performed in accordance with the European Guidelines for Self-Compacting Concrete [30], which means that, despite the negative effect of rubber aggregate on SCC fresh state properties observed, acceptable test results could be obtained with the proper amount of rubber aggregate replacement level.

### 3.2. Porosity/Air Content

Uygunoğlu and Topçu [23] reported an increase in the apparent porosity of self-consolidating mortar mixtures when partially replacing fine aggregate with scrap rubber particles (size 1–4 mm) by volume. With a water to powder ratio (w/p) = 0.47, the increases in apparent porosity were approximately 23%, 29%, 30%, 41% and 41% with inclusion of 0%, 10%, 20%, 30%, 40% and 50% rubber aggregate content, respectively. Ismail and Hassan [7] reported an increase in air content of SCC mixtures by partially replacing fine aggregates with crumb rubber (size 0–4 mm) by volume. The increase in air content was from 1.5% to 6.8% with increasing crumb rubber content from 0% to 40%. AbdelAleem and Hassan [22] partially replaced fine aggregates in SCC mixtures with rubber aggregates (size 0–4.75 mm). Rubber aggregate replacement by volume of fine aggregates was 0%, 5%, 10%, 15%, 20%, 25% and 40%; the increase in air content was from 2.70% to 4.20% for SCC mixture with rubber content 0% and 20%, also containing silica fume (SLF). Another investigation on the influence of different rubber aggregate pre-treatment on mechanical and dynamic properties of SCRC was carried out by Najim and Hall [34]. They reported an increase in apparent porosity by 19% when rubber aggregate was pre-treated with mortar pre-coating technique. Ismail and Hassan [33] reported an increase in air content of SCC mixtures by partially replacing fine aggregates with crumb rubber (maximum size 4.75 mm) at different levels, by volume. Decrease in air content was from 5% to 1.5% with reducing rubber aggregate content from 30% to 0%.

From the test results that are discussed in Section 3.2 and summarized in Table 2, it can be highlighted that with an increase of rubber aggregate in SCC mixtures an increase in porosity occurs. The increase in porosity can be explained well by the same reason as in Section 3.1: rubber particles have rough surface [14] and sharp edges [22], thus entrapping air and increasing the porosity of self-compacting concrete.

### 3.3. Rheological properties

Güneyisi et al. [35] investigated the rheological properties of fresh SCC mixtures with the inclusion of different types of rubber aggregate. They partially replaced fine and coarse aggregate in SCC mixtures with crumb rubber (size 0–4 mm) and tire chips (10 to 40 mm length size) at different levels, by volume of 5%, 10%, 15%, 20% and 25%. Three different sizes of crumb rubber (CR) were used in experimental investigations, size 0–1 mm (size No. 18), 1–4 mm (size No. 5), and 0–4 mm (sizes No. 18 and No. 5) and one size of tire chips (TC) was used in experimental investigation (10 to 40 mm length size). Rheological properties of fresh SCRC mixtures were investigated using a rheometer. During the rheometer testing, torque values for different rotational speed values were determined. As rubber aggregate content increased, an increase in torque value was observed, regardless of rotational speed value. With 25% rubber aggregate replacement and a rotational speed value of 0.050 rps, torque values of different types of rubber aggregate, i.e., size 0–1 mm (size No. 18), 1–4 mm (size No. 5), 0–4 mm (sizes No. 18 and No. 5), and tire chips (10 to 40 mm length size), were 4.2, 5.1, 4.6 and 6.5 times higher, respectively. 

Further, Güneyisi et al. found that the rheological properties of fresh SCRC mixtures were interpreted with two types of models, Herschel-Bulkley model and modified Bingham model. Variations in values of the exponent *n* (Herschel-Bulkley model) and coefficient *c*/*μ* (modified Bingham model) were observed. While considering that values of exponent *n* and coefficient *c*/*μ* for all SCC mixtures with rubber aggregate were greater than one (*n* > 1) and greater than zero (*c*/*μ* > 0), shear thickening behaviour was noticed. Decreasing rubber content in SCC mixture systematically reduced exponent *n* and coefficient *c*/*μ*, which means a greater shear thickening behaviour. The same behaviour occurred with replacement with different types of sizes of rubber aggregate. With rubber aggregate sizes No. 18, No. 5, No. 18 + No. 5, and tire chips, an increase in exponent *n* was from 0.253 to 0.391, 1.054, 0.876 and 1.788, respectively, while the increase in coefficient *c*/*μ* was from 1.135 to 1.205, 1.410, 1.363 and 1.543, with increase in rubber content ranging from 0% to 25%, respectively.

As in Section 3.3, it can be noted that rheological properties of self-compacting concrete with waste tire rubber as partial replacement for natural fine or coarse aggregate still needs further investigation. Due to the lack of experimental investigations and test results to compare, the rheological properties of SCRC should be an area of interest for further laboratory tests because of the importance of understanding the workability properties of this self-compacting concrete type.

## 4. Hardened SCC Properties

### 4.1. Compressive Strength

**Figure 4:**
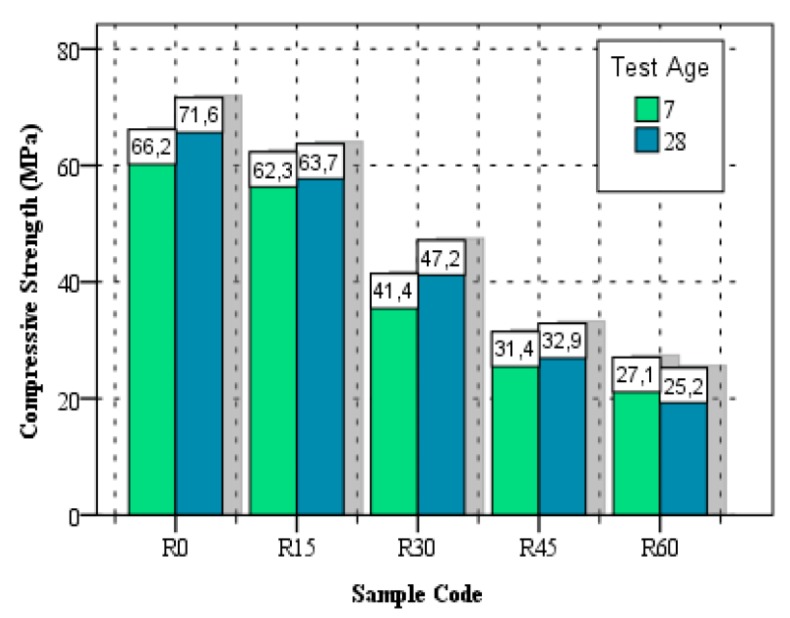
Test results of compressive strength of rubberized SCC [6]. Reproduced with permission from M. Emiroğlu, S. Yildiz, O. Keleştemur, and M. H. Keleştemur, Bond performance of rubber particles in the self-compacting concrete; published by Publisher creation—J. Cairns, G. Metelli, G. Plizzari (eds.), 2012.

Reduction in the compressive strength of SCC is inevitable when different types of rubber aggregate were used as a partial replacement of natural aggregate, regardless of rubber particle size, rubber replacement level, or replaced type of aggregate. In all cases, a reduction in compressive strength was reported and with higher level of rubber replacement, reduction in compressive strength was more pronounced, as shown in Table 3 and Figure 4. This reduction effect can be attributed to poor adhesion and bond strength between rubber particles and cement paste, low rubber modulus of elasticity as compared to natural aggregates and a greater amount of air entrapped between rubber particles and cement paste [5,22]. According to Hilal [36], rubber particles act like voids in the hardened concrete specimens causing a reduction in compressive strength.

However, in several experiments in which supplementary cementing materials (SCMs) were used, enhancements in SCRC compressive strength was reported. Metakaolin (MK) was found to be the best SCM in terms of improvement of SCRC compressive strength [7,37]. When 20% MK was added as a SCMs to SCRC mixtures, the relative increase in compressive strength was 44%, 47% and 56% with 20%, 30% and 40% rubber aggregate contents, respectively [7]. Furthermore, Ganesan et al. [20] observed the influence of steel fibres (SLFs) on compressive strength of an SCRC with 15% rubber content. With inclusion of 0.50% and 0.75% SLFs, a negligible reduction of 3.87% and 1.12% in 28-day compressive strength was reported. Minor relative improvement in SCC compressive strength could also be achieved with pre-treatment of rubber particles with NaOH, according to Si et al. [14]. On the other hand, with fly ash (FA) as a partial replacement for the binder in SCRC, Güneyisi [25] reported even greater reduction in compressive strength when compared to SCRC without FA.

According to the test results that are given in Section 4.1, it can be concluded that it is possible to use waste tire rubber as a partial replacement for natural fine or coarse aggregate and to maintain values of compressive strength (*f*_c_) lower than the limit for structural applications, i.e., *f*_c_ > 17 MPa [39], but the appropriate replacement level must be determined first.

### 4.2. Flexural Strength

In most cases, several authors have reported reduction in flexural strength by implementing waste tire rubber as partial replacement of natural fine or coarse aggregate in SCC. For the same rubber replacement level, flexural strength reduction was similar, irrespective of rubber particle size, as shown in Table 4. This reduction can be explained with the same reasons given in Section 4.1. Entrapped air between rubber particles and cement paste, which appears to be due to the rough rubber surface, has a negative impact on flexural strength [39], just as it has a negative impact on compressive strength. However, some test specimens with rubber did not experience a rapid collapse during the test procedure, most likely due to the elastic behaviour of rubber particles [23]. However, opposite results, an increase in flexural strength with inclusion of waste tire rubber, were also observed, due to the better load carrying capacity of the rubber aggregate [20]. Similar to the conclusion in Section 4.1, the enhancement of flexural strength of rubberized self-compacting concrete is possible by using different kind of supplementary cementing material, i.e., metakaolin (MK), which can improve 7-day and 28-day flexural strength of SCRC specimens, and in that way significantly mitigate the decrease of flexural strength [37]. With 20% MK as SCMs in SCRC mixtures, the relative increase in flexural strength was 18%, 5% and 11% with inclusion of 20%, 30% and 40% CR content, respectively [7].

### 4.3. Splitting Tensile Strength

From the test results of different authors, it can be undoubtedly noted that there is a reduction in the splitting tensile strength with increased amount of rubber aggregate in test specimens, regardless of rubber particle size, replacement level, or replaced material, as shown in Table 5. The rubber replacement levels tested were up to 50% [7], and the highest reduction in splitting tensile strength happened at this replacement level. As in Section 4.1 and Section 4.2, this mechanical property of SCC could be enhanced by SCM [37] or with rubber pre-treatment methods [14]. Najim and Hall [34] reported increase in 28-day splitting tensile strength of SCRC specimens by 19% when the rubber aggregates were pre-treated with a mortar pre-coating method. Addition methods of enhancement in splitting tensile strength can be achieved with adding larger length fibres, which positively affected splitting tensile strength values [28].

### 4.4. Modulus of Elasticity

Replacement of natural fine (FA) and coarse aggregate (CA) with rubber aggregate had a negative influence on the static and dynamic modulus of elasticity. The static and dynamic modulus were reduced with an increasing rubber replacement level. This concrete behaviour can be attributed to air entrainment caused by rubber particles and lower modulus of elasticity of rubber aggregate than natural fine or coarse aggregate modulus [39]. Rubber particles have a poor bond with cement paste, and therefore act like voids in the concrete [36]. Additionally, under compressive load, rubber particles are separated from the cement matrix and make failure easier [27]. However, a relative increase in the modulus of elasticity was reported when the rubber particles were pre-treated [34] or when metakaolin was used as supplementary cementing material [7]. Test results from different authors of the influence of rubber aggregate on elastic modulus are given in Table 6.

### 4.5. Impact Resistance

**Figure 5:**
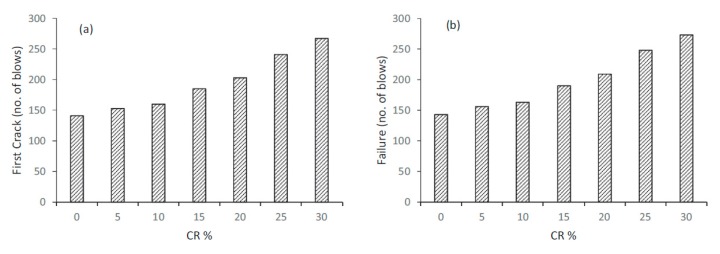
Impact resistance of self-compacting rubberized concrete under drop weight test: (**a**) effect of crumb rubber on N1; (**b**) effect of crumb rubber on N2 [28]. Reproduced with permission from B.H. AbdelAleem, M.K. Ismail, and A.A.A. Hassan, The combined effect of crumb rubber and synthetic fibers on impact resistance of self-consolidating concrete; published by Elsevier, Construction and Building Materials. 2018.

The influence of rubber aggregate on impact resistance of SCC was studied by AbdelAleem et al. [28]. They reported that incorporating crumb rubber in SCC mixtures enhanced the impact resistance of concrete in both drop-weight impact resistance tests (number of blows) and in flexural loading impact resistance tests (impact energy). Drop-weight test results are presented in Figure 5, and they showed an increase in the number of blows for ultimate failure (N2) up to 91% and in number of blows for first crack (N1) up to 89% when the percentage of crumb rubber varied from 0% up to 30%, whilst the flexural loading test results showed an increase in ultimate impact energy of up to 2.42 times when the percentage of crumb rubber varied from 0% to 25%. Furthermore, adding fibres to SCC mixtures notably affected enhancement of impact resistance.

Khalil et al. [29] also investigated the influence of rubber aggregate inclusion on impact resistance of SCC. Drop-weight test results showed an increase in N2 and in N1 by 2.8 and 3 times, i.e., 179% and 200%, respectively, when the percentage of crumb rubber varies from 0% to 30%. Ismail and Hassan [33] reported improvement in impact resistance of SCC containing rubber aggregate content in drop-weight impact resistance test (number of blows) and in flexural loading impact resistance test. Flexural loading test results showed an increase in N2 by 21%, 68%, 101%, 129%, 142% and 116% with the inclusion of 5%, 10%, 15%, 20%, 25% and 30% crumb rubber, respectively. Drop-weight test results showed an increase in N2 and N1 from 143 and 141 to 273 and 267, respectively, when increasing the rubber content from 0% to 30%, respectively.

As a result of an insufficient number of experimental investigations carried out on impact resistance of SCRC, several investigations of impact resistances of rubberized concrete (RC) are given below, in order to compare the two concrete types and conclude whether similarities or between them.

Pedro et al. [40] reported an increase in impact resistance of concrete specimens containing rubber aggregate instead of fine aggregate. They reported an increase in the height of the rupture when rubber aggregate was used as partial replacement; the dent diameter of rubberized concrete was between 1.67 and 2 times larger than in traditional concrete. Miller and Tehrani [4] reported no significant tendency between determined maximum net impulse and rubber aggregate content. Vadivel et al. [41] partially replaced fine aggregate, coarse aggregate, and fine-coarse aggregate with rubber crumb and rubber chips (size 0–20 mm). They reported increases and decreases in impact resistance with inclusion of rubber aggregate content. Drop-weight test results showed an increase in N1 and N2 numbers by 15% and 19% when fine and coarse aggregate were partially replaced with 6% rubber aggregate content overall (3% fine + 3% coarse). On the other hand, test results showed a reduction in the same values by 18% and 19% when fine aggregate was replaced with 6% rubber content and by 42% and 39% when coarse aggregate was replaced with 6% rubber content.

Thus, to summarize, the inclusion of rubber particles in SCC or traditional concrete appears to cause enhancement in impact resistance. This performance can be attributed to the low stiffness of rubber particles, which causes increase in post-cracking resistance and enhances the ductility of the SCC specimens [28,33]. However, there are still many unknowns in the behaviour of SCRC and its impact resistance, hence further experimental investigations are worthy to pursue due to the possible application of SCRC in concrete structures.

### 4.6. Ductility and Brittleness

**Figure 6:**
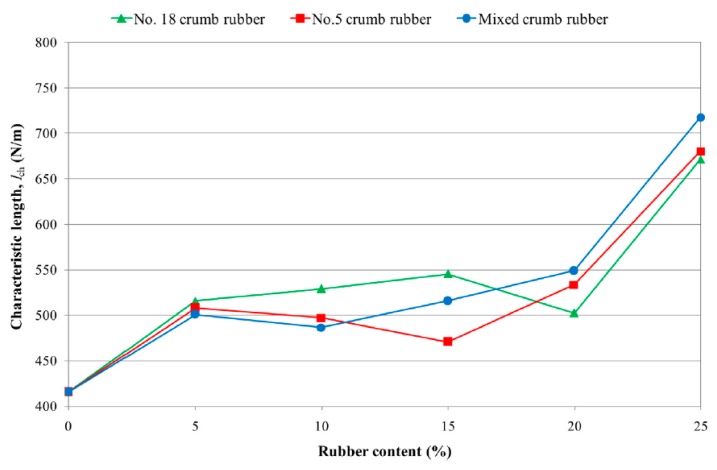
Relationship between characteristic length and rubber replacement level [36]. Reproduced with permission from Hilal, N.N. Hardened properties of self-compacting concrete with different crumb rubber size and content; published by Elsevier, International Journal Sustainable Built Environment, 2017.

Hilal [36] reported an increase in ductility of SCC specimens by partially replacing fine particles in SCC mixtures with rubber particles (size 0–4 mm). As can be seen in Figure 6, their results showed an increase in characteristic length by 20.55%, 17.11%, 24.07%, 32.07% and 72.74% when fine aggregates were replaced with 5%, 10%, 15%, 20% and 25% crumb rubber (size 0–4 mm) by volume, respectively.

Due to lack of experimental results, this hardening property of SCC with waste tire rubber still needs more investigation. However, other authors also investigated the ductility of traditional concrete with waste tire rubber replacing fine or coarse aggregate and results of these authors are given below. Vadivel et al. [41] reported an increase in ductility with inclusion of rubber aggregate content. The ductility index, i.e., the ratio of energy consumed at ultimate failure and energy consumed at first crack of drop weight test, were 6.4% and 2.7% higher when 6% of coarse and 6% of fine/coarse aggregate, respectively, were partially replaced with rubber crumb and chips. Gesoğlu et al. [8] investigated the influence of different types and particle sizes of rubber aggregate on concrete ductility. They reported an enhancement in ductility when fine crumb rubber (1.00–4.00 mm) was used, while a reduction in ductility was reported if tire chips (size up to 10 mm) or very fine crumb rubber (size 0.1–1.00 mm) were used. The maximum enhancement in characteristic length was 168% with inclusion of 20% fine crumb rubber, while the maximum reduction was 62% with inclusion of 10% tire chips and 10% very fine crumb rubber. Youssf et al. [42] also studied the influence of crumbed scrap tyre rubber (size 1.18, 2.36 mm, and 0.15 to 2.36 mm) in concrete with additional confinement being obtained with fibre reinforced polymer (FPR). They partially replaced fine aggregate with crumb rubber by 0% to 20% of fine aggregate volume and reported enhancement of concrete ductility with increasing rubber aggregate content by volume of fine aggregate and with an increasing confinement level. Increase in concrete ductility, which was defined as concrete ultimate strain and concrete yield strain ratio, was 42% with the inclusion of 20% well graded rubber aggregate content, with one confinement layer being used in both concrete samples.

Zheng et al. [43] reported improvement in ductility when coarse aggregate was replaced with rubber powder or tire chips. The reduction in brittleness index, which was defined as a ratio of the reversible (elastic) to irreversible (plastic) deformations, was 45%, 52% and 54% when coarse aggregate was replaced with 15%, 30% and 45% by volume with rubber powder, respectively, and 27%, 46% and 64%, respectively, with same replacement level with tire chips. Elghazouli et al. [12] investigated the influence of rubber content on the ductility of rubberized concrete specimens. Rubber aggregate particle size varied from 0 to 20 mm with replacement levels of 45% and 60%. Test results showed enhancement in ductility with increase of rubber aggregate content. Improvement in concrete rotational capacity was up to 50% compared to concrete without rubber aggregate, which contributed to appropriate and desirable ductile crushing behaviour of rubberized concrete under cyclic loading. Gesoğlu et al. [44] reported improvement in concrete ductility when rubber aggregate was added as replacement for natural aggregate. With only 5% rubber aggregate, the characteristic length was approximately 1.85 times higher than the control specimen without rubber aggregate. Similar results were obtained when the replacement level was higher than 5%, except the results with a mix of crumb rubber and tire chips showed even greater enhancement in concrete ductility with increasing rubber aggregate content, approximately 3.4 times higher values with the inclusion of 20% rubber content.

The test results that are analysed above are summarized in Table 7. In order to firmly conclude about the influence of rubber implantation in SCC, further investigation needs to be done, due to the lack of test results on the ductility of SCRC. However, results from authors listed in Table 7 are promising, due to the fact that increase of ductility of rubberized concrete (RC) was shown in most of the experimental investigations. With appropriate replacement levels of waste tire rubber and rubber particle size, an improvement in SCC ductility could be achieved.

### 4.7. Fracture Energy

Hilal [36] reported a reduction in the fracture energy (GF) of SCC specimens by partially replacing fine particles in SCC mixtures with rubber particles (size 0–4 mm). The results showed a reduction in fracture energy by 10.91%, 13.03%, 18.55%, 24.01% and 28.05% when fine aggregate was replaced with 5%, 10%, 15%, 20% and 25% crumb rubber (size 0–4 mm) by volume, respectively. The results of fracture energy testing carried out by Bideci et al. [24] indicated that with inclusion of 10% and 15% of rubber aggregate content in SCC with length size of 50 mm, there was an increase in fracture energy of 1% and 5%; in all other cases, they reported a reduction in fracture energy by a maximum of 35%. Due to the lack of experimental results, it can be noted that detailed study of fracture energy of SCRC still needs to be done. To better understand or to predict SCRC behaviour and its fracture energy, several test results from authors who investigated the fracture energy of traditional concrete with waste tire rubber are given below.

**Figure 7:**
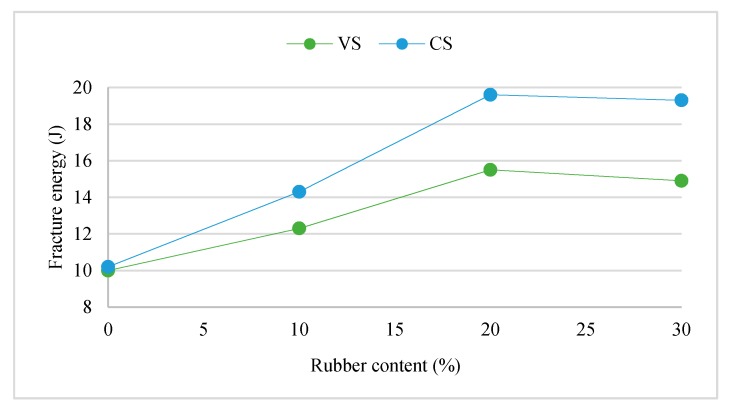
Relationship between fracture energy and rubber replacement level [45]. Reproduced with permission from A. Moustafa, M. ElGawady, Dynamic Properties of High Strength Rubberized Concrete; published by American Concrete Institut (ACI), Special Publication, 2017.

Gesoğlu et al. [8] reported a reduction and enhancement in fracture energy of pervious concrete depending on the type and particle size of rubber aggregate. The fracture energy decreased by 25% and 74% with the inclusion of 20% very fine crumb rubber and 20% mix of tire chips and very fine crumb rubber, respectively. However, the fracture energy increased up to 42% with inclusion of 10% tire chips or 20% mix of tire chips and fine crumb rubber. Moustafa and ElGawady [45] investigated the dynamic properties of high performance concrete with rubber aggregate. As shown in Figure 7, they reported a decrease of concrete fracture energy with a decrease of rubber aggregate content. The increase in fracture energy of constant slump (CS) concrete mixture with 30% rubber replacement was 85.2%. Furthermore, they reported that the mid-span deflection of rubberized concrete was larger than that of traditional concrete.

Gesoğlu et al. [44] tested two types of scrap tire rubber, crumb rubber (size 0–4 mm) and tire chips (size 10–40 mm), individually and mixed. Replacement level was 5%, 10%, 15%, 20%, 25% and 30% by volume of fine or coarse aggregates. Regardless of rubber type, an increase in fracture energy occurred up to a replacement level of 15%. The enhancement in fracture energy was up to 35% with 15% of crumb rubber. However, 20%, 25% and 30% replacement levels showed a smaller enhancement in fracture energy, approximately 26%, 21% and 20%, respectively, when crumb rubber was used.

From the above, it can be concluded that test results from both types of rubberized concrete, SCC and traditional concrete, gives scattered results. Increases and decreases in fracture energy were reported, and thus it cannot be clearly concluded whether rubber inclusion improves or reduce fracture energy. However, with a proper amount and rubber particle size, fracture energy can be improved, and because of this, further investigations on fracture energy of SCRC are promising and should be carried out.

### 4.8. Water Absorption and Water Sorptivity

**Figure 8:**
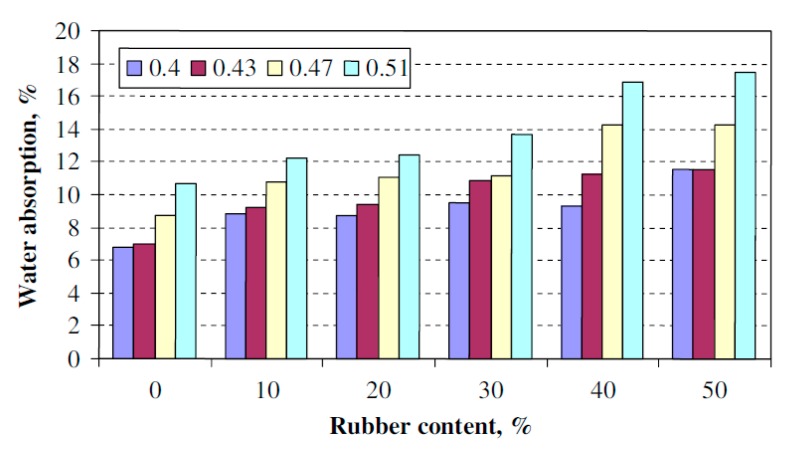
Effect of rubber aggregate content on water absorption of self-consolidating mortar [23]. Reproduced with permission from T. Uygunoğlu and I.B. Topçu, The role of scrap rubber particles on the drying shrinkage and mechanical properties of self-consolidating mortars; published by Elsevier, Construction and Building Materials 2010.

Uygunoğlu and Topçu [23] reported an increase in water absorption (WA) of self-consolidating mortar specimens by partially replacing fine aggregate with scrap rubber particles (size 1–4 mm) by volumes of 0%, 10%, 20%, 30%, 40% and 50%. As shown in Figure 8, four different water to powder ratios were used, 0.4, 0.43, 0.47 and 0.51. When the water to powder ratio increased, water absorption also increased. A similar behaviour occurred when rubber content increased. With w/p = 0.4, increase in water absorption was approximately 38%, 38%, 46%, 43% and 77% with inclusion of 0%, 10%, 20%, 30%, 40% and 50% rubber aggregate content, respectively.

Bideci et al. [24] reported an increase in water absorption of SCC specimens with an increase of rubber aggregate (RA) content obtained from waste bladder tyres. With inclusion of up to 15% rubber aggregate content in SCC mixtures, the increase in water absorption was up to 88% for different rubber aggregate length sizes (25, 50 and 75 mm). Bignozzi and Sandrolini [27] partially replaced the natural fine aggregate in SCC mixtures with untreated tyre rubber (TR) aggregates by volumes of 0%, 22.2% and 33.3%. They reported a small increase in water absorption (WA) with an increasing rubber content in SCC specimens. The enhancement in water absorption was 4% and 10% with the inclusion of 22.2% and 33.3% tyre rubber, respectively.

Gesoğlu and Güneyisi [46] reported an increase in water absorption by partially replacing fine aggregate in SCC mixtures with crumb rubber content (size 0–4 mm) by volumes of 0%, 5%, 15% and 25%. Increase in 28-day and 90-day water absorption was approximately 3%, 11%, 34% and 3%, 8%, 29% with the inclusion of 5%, 15% and 25% rubber aggregate content, respectively. However, when greater amounts of fly ash (FA), i.e., 40% or 60%, were combined with the inclusion of crumb rubber, water absorption reduced. Furthermore, Gesoğlu and Güneyisi [46] reported an increase in water sorptivity by partially replacing fine aggregate in SCC mixtures with crumb rubber content (size 0–4 mm) by volumes of 0%, 5%, 15% and 25%. Increase in the 28-day sorptivity coefficient was approximately 5.26%, 25%, 39.47% and the 90-day sorptivity coefficient 10%, 29%, 43% with the inclusion of 5%, 15% and 25% rubber aggregate content, respectively.

For comparison purposes, several test results from different authors of the water absorption of traditional concrete with waste tire rubber are given below. Bjegović et al. [47] investigated the influence of partially replacing natural fine aggregate in conventional concrete with different sizes of rubber aggregate (size 2–3.5 mm, 2–4 mm, and 0.5–2 mm), by volumes of 5%, 10% and 15%. They reported reduction in water absorption by 78% with increasing rubber aggregate content from 5% to 15%. Gupta et al. [48] partially replaced fine aggregates with rubber ash particles (size 0.15–1.9 mm) derived from a pyrolysis technique and with rubber fibres (size 2–3 mm width and maximum 20 mm length) derived from mechanical grinding. For a water to cement ratio (w/c) = 0.45, increase in water absorption was 17% and 4% with the inclusion of 20% rubber ash content and 25% rubber fibres, respectively. Kumar et al. [49] reported an improvement in water absorption by partially replacing fine and coarse aggregate with rubber powder and chipped rubber. Increase in water absorption was 45%, 29%, 26% and 23% with the inclusion of 40%, 30%, 20% and 10% rubber aggregate content, respectively. Thomas et al. [50] reported an increase in water absorption with increasing rubber aggregate content in concrete. When compared to control specimens (0% replacement level), water absorption was up to two times higher, with a 20% replacement level. The results of water absorption test carried out by Pedro et al. [40] showed that with a 15% inclusion of rubber aggregate, water absorption is higher by 3.84% and 11.62% when mechanically shredded rubber and cryogenic rubber were used as substitute aggregate, respectively.

From the above, it can be remarked that the inclusion of waste tire rubber in both types of concrete, SCC and traditional concrete, causes an increase in water absorption. This behaviour can be attributed to entrapment of air of interfacial transition zone (ITZ) between rubber particles and cement paste [23,27], and to some deviations of rubber particles, i.e., rough surface of rubber particles [46]. Furthermore, an increase in water absorption can be achieved by increasing the water to powder ratio [23].

### 4.9. Shrinkage

**Figure 9:**
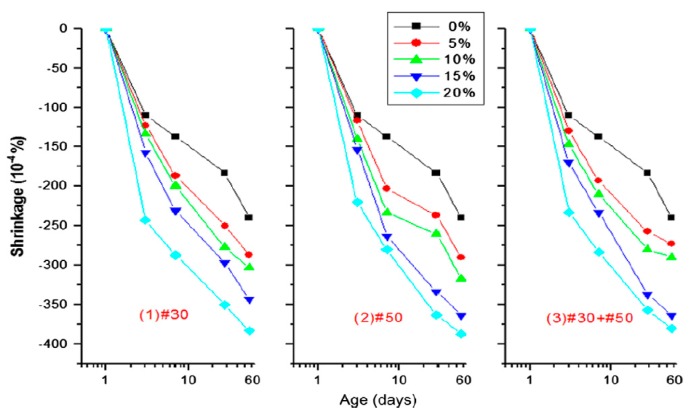
Effect of rubber aggregate on shrinkage [38]. Reproduced with permission from W. H. Yung, L. C. Yung, and L. H. Hua, A study of the durability properties of waste tire rubber applied to self-compacting concrete; published by Elsevier, Construction and Building Materials, 2013.

Uygunoğlu and Topçu [23] reported an increase in shrinkage of self-consolidating mortar specimens by partially replacing fine aggregate with scrap rubber particles (size 1–4 mm) by volumes of 0%, 10%, 20%, 30%, 40% and 50%. With w/p = 0.51, increase in 180-day shrinkage was approximately 1.74, 1.83, 1.96, 3 and 4 times higher with the inclusion of 0%, 10%, 20%, 30%, 40% and 50% rubber aggregate content, respectively. Furthermore, they reported increase in shrinkage by increasing the water to powder ratio. Si et al. [14] partially replaced fine aggregate in SCC mixtures with crumb rubber granules (size 1.44–2.83 mm). Crumb rubber replacement, as measured by replacement volume of sand, was 0%, 15% and 15% for NaOH pre-treated rubber and 25% NaOH pre-treated rubber. They reported an increase in shrinkage with increasing rubber content. The reduction alkali silica reaction (ASR) was 24% and 7% with the inclusion of 25% NaOH treated rubber content, 15% NaOH treated rubber content and 15% untreated rubber content, respectively. Yung et al. [38] reported shrinkage increases of SCC specimens by partially replacing fine aggregate (sand) with tire rubber powder (size #30, #50), by volumes of 0%, 5%, 10%, 15% and 20%. Average length change was 35% higher than the length change of the control specimen with inclusion of 5% rubber powder content. As rubber powder content became higher, the average length change was greater. With inclusion of 20% rubber powder content, average length chance was 95% higher than length change of the control specimen (Figure 9).

Another investigation on shrinkage of SCC with rubber aggregate was carried out by Zaoiai et al. [21]. They partially replaced the natural fine and coarse aggregate with rubber aggregate (size 0/3 and 3/8) at different levels and reported a reduction in 28-, 90-, 200-, and 300-day shrinkage when increasing rubber aggregate content. Shrinkage reduction was most expressed after 300 days. The results showed that after 300 days, reduction in shrinkage was 16.09%, 33.30%, 19.79% and 59.47% with the inclusion of 2.5%, 5%, 10%, and 20% rubber aggregate content, respectively.

Shrinkage can be crucial factor for the design of structural members because of the length variation in time [21], and due to this reason, in order to compare a larger number of different test results and to reliably understand the influence of rubber incorporation on concrete shrinkage, several test results from different authors of shrinkage of traditional concrete with waste tire rubber are given below.

Turatsinze et al. [51] observed concrete shrinkage behaviour in ring testing by replacing fine aggregate with rubber. The test time was 55 days and they reported the reduction in concrete shrinkage when rubber aggregate was incorporated in concrete. With 30% of rubber aggregate inclusion, the time to first crack initiation was prolonged by 2.83 times, the number of cracks was reduced by four times, main crack length was reduced by 36%, and maximum crack opening was reduced by 90%. Another investigation on shrinkage behaviour was carried out by Pedro et al. [40]. They reported an increase in shrinkage when the rubber aggregate replaced fine aggregate by 15% of volume. 90-days shrinkage was approximately 40% and 63% higher when mechanically shredded rubber and cryogenic rubber were used as the replacement. Bravo and de Brito [52] replaced natural fine and coarse aggregate with tyre rubber aggregate (size less than 4 mm and less than 11.2 mm) by total volume of natural aggregate, by different replacement levels of 5%, 10% and 15%. Different types of tyre aggregate obtained by cryogenic or mechanical grinding were used as well. They reported increase in shrinkage with increasing rubber aggregate volume in the concrete mixture. Increase in 90-days shrinkage was approximately 45% higher with fine aggregate replaced with rubber aggregate at a replacement level of 15%.

From the above, it can be noted that shrinkage of SCC and traditional concrete becomes higher as more waste tire rubber was added in concrete, regardless to rubber particle size. Shrinkage is in direct link with water to powder ratio. As the water to powder ratio increases, porosity is also increasing, causing an increase in concrete shrinkage [23]. Nevertheless, the inclusion of waste tire rubber in SCC causes increases in shrinkage, which is probably due to elasticity modulus, i.e., stiffness of rubber particles that is much lower than natural aggregate, causing larger deformation while the cement matrix shrinks [14,23].

### 4.10. Fatigue Behaviour 

Ganesan et al. [20] studied the influence of the inclusion of rubber aggregate content in SCC on the fatigue performance of SCC. Fatigue behaviour was observed, as measured by two factors, maximum stress level (*S*), which ranged from 90% to 60% of the static flexural strength, and number of cycles to failure (*N*). An increase in fatigue life was reported when a greater amount of rubber content was included in the SCC mixtures. At a stress level *S* = 0.90, *N* increased by the same value of 1.44 times for both SCRC mixtures with 15% and 20% rubber content. At a stress level *S* = 0.60, *N* increased by approximately the same value, 1.62 and 1.63 times for both SCRC mixtures with 15% and 20% rubber content.

**Figure 10:**
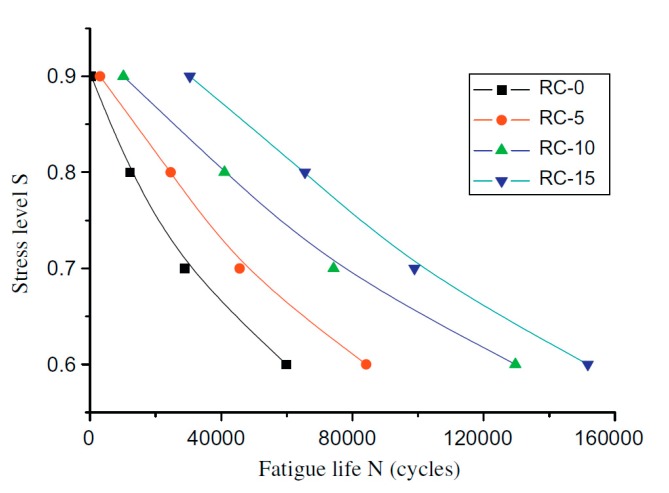
Relationship between fatigue life (N) and stress level (S) of rubberized concrete [53]. Reproduced with permission from F. Liu, W. Zheng, L. Li, W. Feng, and G. Ning, Mechanical and fatigue performance of rubber concrete; published by Elsevier, Construction and Building Materials, 2013.

Due to a lack of experimental data of fatigue behaviour of self-compacting rubberized concrete, several test results of rubberized concrete and its fatigue resistance are given below. Liu et al. [53] replaced fine aggregate with rubber aggregate (size 2 mm) by volumes of 0%, 5%, 10% and 15%. They reported a decrease in *N* with an increase in maximum stress level (*S*). However, the increase in *N* was reported when rubber aggregate had been included in concrete with constant stress level (*S*). At a stress level *S* = 0.90, *N* increased by approximately 7, 11, and 32 times with 5%, 10% and 15% rubber aggregate content, respectively. Similar results were obtained with lower level of stress (*S*), i.e., 0.60, 0.70 and 0.80, as is shown in Figure 10. 

Analogous test results were reported in another experimental investigation that was carried out by Murugan et al. [16]. They partially replaced fine aggregate with waste tire crumb rubber of volumes of 5%, 10%, 15%, 20% and 25%. Fixed water to cement ratio (0.32) and amounts of superplasticizer (SP) were used during experiments. Improvement in fatigue concrete behaviour was reported at up to 10% rubber aggregate replacement. With inclusion of 5% and 10% of crumb rubber, the increase in flexural fatigue performance was 1.46% and 1.96%, respectively. Furthermore, average *N* also increased with inclusion of rubber aggregate content. At a stress ratio *S* = 0.80, *N* was 3.15, 2.95 and 1.62 times higher with 5%, 10%, and 15% rubber content, respectively. Mohammadi et al. [54] investigated the influence of integrating crumb rubber in concrete on fatigue behaviour while using the cyclic loading test. With constant water to cement ratio of 0.40 and stress level 0.95, *N* increased by approximately 19% when 40% of natural aggregate was replaced with crumb rubber. In all other replacement cases, with 10%, 20% and 30% inclusion of crumb rubber, they reported negative or no impact on fatigue behaviour, i.e., *N*.

From the above it can be concluded that regardless of type of concrete, replaced aggregate material or rubber particles size, enhancement in fatigue resistance of concrete and self-compacting concrete is inevitable with addition of waste tire rubber. Although, further investigation on self-compacting rubberized concrete should be carried out in order to give a better explanation of influence of rubber particle size and replacement level on fatigue behaviour.

### 4.11. Interfacial Transition Zone (ITZ) and Scanning Electron Microscope (SEM)

**Figure 11:**
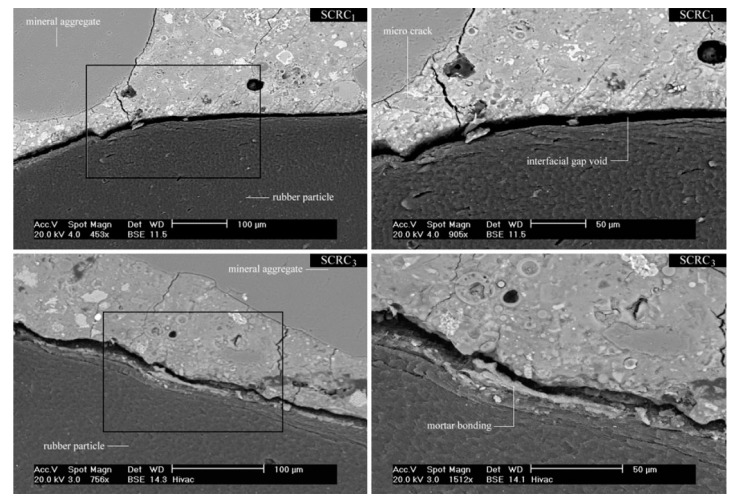
Interfacial bonding between cement paste and rubber aggregate without pre-treatment (SCRC1) and with pre-coating with mortar—(SCRC3) [34]. Reproduced with permission from K. B. Najim and M. R. Hall, Crumb rubber aggregate coatings/pre-treatments and their effects on interfacial bonding, air entrapment and fracture toughness in self-compacting rubberised concrete (SCRC); published by Elsevier, Materials and Structures, 2013.

Interfacial bonding between cement matrix and rubber aggregate is one of the most important factors that affect almost every SCC hardened state property. Bignozzi and Sandrolini [27] reported effective adhesion between tire rubber and cement matrix in cases when the rubber particles were previously pretreated and covered with cement matrix. In another investigation carried out by Emiroğlu et al. [6], interfacial bonding and stress transfer between fibres and the cement matrix was explained with mechanical interlocking of two materials, due to the rough surface of rubber aggregate. Furthermore, they reported enhancement in interfacial transition zone between rubber aggregate and cement paste when higher amounts of finer materials, such as supplementary cementitious material, were used. Najim and Hall [34] used different pre-treatment methodologies for rubber, such as NaOH pre-treatment, cement paste coating, water washing, and mortar coating. After a mixing procedure, microstructural analysis and porosity was determined by using scanning electron microscopy (Figure 11).

When compared to untreated rubber, an increase in compressive strength and splitting tensile strength was reported by 37 and 19% respectively, when rubber particles were pre-coated with mortar, which caused a significant improvement in the interfacial bonding and hence improved the stress transformation.

Only a small number of investigations have focused on the investigation of ITZ of self-compacting rubberized concrete and on investigation of ITZ while using SEM, although it is obvious that bonding between cement paste and rubber particles is a crucial factor to develop reliable self-compacting rubberized concrete with desirable mechanical properties. Therefore, future investigations of SCRC on SEM still needs to be done, all for finding an optimal solution of bonding between rubber particles and cement matrix.

## 5. Discussion

In the present study, the influence of recycled waste tire rubber as a fine and/or coarse natural aggregate replacement in SCC on its fresh and hardened properties was reviewed. See Table 8.

Overviews of each fresh and hardened property of SCRC as tested and analysed in the literature and several general conclusions can be made:

- Increase of waste tire rubber negatively effects fresh SCC properties. Flowability, passing ability, and segregation resistance were reduced and viscosity was increased, although a small number of authors claim the opposite. This can be attributed to an increase of rough surface and surface friction of rubber particles. Only a few authors replaced natural aggregate with waste tire rubber above level of 40%, but it can be determined that, in order to maintain acceptable fresh SCC properties, replacement level should not exceed 20%. Furthermore, most authors used different kinds of chemical admixtures to maintain or even to improve fresh SCC properties, i.e., superplasticizer (SP), viscosity modified admixture (VMA), or high range water reducer admixture (HRWRA). Different pre-treatment methods were used by several authors in order to improve the bond connections between rubber aggregate and cement matrix. Water soaking method was shown to be a cost-effective method. Most of the test results of flowability, viscosity, passing ability, and segregation resistance were within the limits given by the European Guidelines for Self-Compacting Concrete.
- An increase in porosity and air content were detected with the increase of rubber aggregate content in SCC mixtures. As for the other fresh concrete properties that are mentioned above, the same explanation can be used, rubber particles have sharp edges and rough surfaces and therefore entrap air in interfacial transition zone (ITZ) between rubber particles and cement paste and increase the porosity of SCC.
- Due to lack of experimental investigations and test results further investigation still needs to be done to determine rheological properties of SCRC in order to better understand the workability properties of this type of SCC.
- Reduction in compressive strength, flexural strength (FS) and splitting tensile strength (STS) of SCC was observed when different types of rubber aggregate were used as a partial replacement of natural fine and coarse aggregate, regardless of rubber replacement level or replaced type of material. This effect can be described as being due to poor adhesion and bond strength between rubber particles and binder content, low rubber modulus of elasticity when compared to natural aggregates and a greater amount of air entrapped between rubber particles and cement paste. However, adding different types of supplementary cementing materials (SCMs), these properties of SCRC can be relatively improved. Also, with the rubber pre-treatment methods, compressive strength, flexural strength (FS) and splitting tensile strength (STS) can be improved but on a smaller level.
- According to test results obtained in this article, SCRC could be used in some structural applications, but the replacement level must be determined rationally in order to preserve acceptable values of mechanical properties, i.e., compressive strength, splitting tensile strength and flexural strength.
- Replacement of natural fine and coarse aggregate with rubber aggregate had a negative influence on static and dynamic modulus of elasticity. Static and dynamic modulus were reduced with increasing rubber replacement levels. This effect can be explained with air entrainment on interfacial transition zone (ITZ) between rubber particles and cement paste and lower modulus of elasticity of rubber aggregate than natural aggregate modulus. Although, a relative increase in modulus of elasticity was reported when the rubber particles were pre-treated or when supplementary cementing materials (SCMs) were used in mixtures, especially metakaolin (MK).
- Enhancement in impact resistance of SCC and traditional concrete occurred with inclusion of waste tire rubber. However, further investigation of SCRC needs to be done to understand the possible applications of SCRC in concrete structures.
- Due to the lack of test results of ductility of *SCRC* and due to the fact that increase in ductility of traditional concrete occurs when rubber was added as replacement for natural aggregate, further investigation of SCRC ductility needs to be done.
- Although scattered results were reported, fracture energy of SCC can be improved with the inclusion of rubber aggregate. Thus, further investigation should be carried out.
- Waste tire rubber increases water absorption of both type of concrete, SCC and traditional concrete, which can be attributed to the rough surface of rubber particles. Furthermore, an increase in water absorption can also be achieved by increasing the w/c ratio.
- Rubber aggregate increases shrinkage of SCC and traditional concrete. Shrinkage becomes higher with higher natural aggregate replacement level.
- Enhancement in fatigue resistance of concrete and self-compacting concrete is inevitable with the addition of waste tire rubber, regardless of type of concrete, replaced aggregate material, or rubber particles size. However, further investigation on self-compacting rubberized concrete should be carried out in order to give reliable explanation of influence of rubber particle size and replacement level on fatigue behaviour.
- Improvement in bonding between cement matrix and rubber particles can be achieved with proper pre-treatment method and can be quantified by using SEM. Still, future investigations of SCRC on SEM needs to be done to find an optimal solution for bonding between rubber particles and the cement matrix.

## 6. Conclusions

The main objective of this review article was a literature overview of fresh and hardened properties of self-compacting concrete with partially replaced natural fine and/or coarse aggregate with recycled aggregate material. From this, it can be concluded:

- Waste tire rubber can be used as a replacement aggregate material in self-compacting concrete.
- Many scientists conducted their experimental work by replacing fine aggregate with rubber aggregate, likely because of the better results that were obtained from experiments with fine aggregate replacement as compared to results obtained with coarse aggregate replacement.
- On behalf of future investigations, it can be suggested that experimental investigations of concrete properties should be investigated with fine aggregate replacement, perhaps with even smaller rubber particles such as waste tire powder.
- From relationship between overviewed fresh and hardened properties of concrete with number of analysed test results for each property depending on concrete type it can be concluded that further experimental work on properties of self-compacting rubberized concrete still needs to be conducted, because of its high potential to be used in structural applications.

## Figures and Tables

**Table 1:** Flowability, viscosity, passing ability, and segregation resistance of self-compacting concrete with increasing of rubber aggregate content.

References	Rubber Sze (mm)	Replacement Level (%)	Replaced Material	Flow-Ability	Viscosity	Passing Ability	Segregation Resistance
Uygunoğlu and Topçu [23]	1–4	10, 20, 30, 40 and 50	FA by volume	reduced	increased	-	-
Ganesan et al. [20]	0–4.75	15 and 20	FA by volume	slightly reduced	increased	slightly increased	-
Emiroğlu et al. [6]	5–12	15, 30, 45 and 60	CA by weight	reduced	-	-	-
Ismail and Hassan [7]	0–4	5, 10, 15, 20, 25, 30, 40 and 50	FA by volume	reduced	increased	reduced	reduced
Bideci et al. [24]	length 75, 50 and 25	5, 10 and 15	CA by volume	reduced	-	reduced	reduced
Aslani et al. [5]	2, 5 and 10	10, 20, 30 and 40	FA and CA by volume	reduced	increased	reduced	-
Güneyisi [25]	0–4	5, 15 and 25	FA by volume	reduced	increased	reduced	-
AbdelAleem and Hassan [22]	0–4.75	5, 10, 15, 20, 25 and 40	FA by volume	reduced	increased	reduced	reduced
AbdelAleem et al. [28]	0–4.75	5, 10, 15, 20, 25 and 30	FA by volume	reduced	increased	reduced	reduced
Rahman et al. [1]	1–4	28	FA	reduced	increased	reduced	-
Turatsinze and Garros [26]	4–10	10, 15, 20 and 25	CA by volume	reduced	-	-	reduced
Bignozzi and Sandrolini [27]	0.5–2.0 0.05–0.7	22.2 and 33.3	FA by volume	reduced	-	reduced	-
Mishra and Panda [19]	5 and 10	5, 10, 15 and 20	CA by weight	increase	reduced	increased	-
Si et al. [14]	1.44–2.83	15 and 25	FA by volume	reduced	increased	reduced	-
Topçu and Bilir [31]	0–4	8, 16.9 and 26.87	FA by weight	reduced	increased	-	reduced
Khalil et al. [29]	0–2	10, 20, 30 and 40	FA by volume	reduced	-	reduced	-
Zaoiai et al. [21]	0/3 or 3/8	5 and 20	FA and CA	reduced	-	increased	reduced
Aslani et al. [32]	5–10	20	CA by volume	reduced	increased	reduced	-
Ismail and Hassan [33]	0–4.75	5, 10, 15, 20, 25 and 30	FA by volume	slightly reduced	increased	reduced	reduced

Note: FA—fine aggregate, CA—coarse aggregate.

**Table 2:** Relationship between rubber aggregate replacement and porosity/air content in self-compacting concrete.

References	Rubber Size (mm)	Replacement Level (%)	Replaced Material	Porosity/Air Content
Uygunoğlu and Topçu [23]	1–4	0, 10, 20, 30, 40 and 50	FA by volume	increase
Ismail and Hassan [7]	0–4	0, 5, 10, 15, 20, 25, 30, 40 and 50	FA by volume	increase
AbdelAleem and Hassan [22]	<4.75	0, 5, 10, 15, 20, 25 and 40	FA by volume	increase
Ismail and Hassan [33]	<4.75	0, 5, 10, 15, 20, 25 and 30	FA by volume	increase
Najim and Hall [34]	2–6	12	FA and CA by weight	increase

**Table 3:** Influence of rubber size, replacement level and replaced material on 28-days compressive strength of self-compacting concrete (SCC).

References	Rubber Size (mm)	Replacement Level (%)	Replaced Material	Initial Value (MPa)	Reduction (%)
Ganesan et al. [20]	0–4.75	15 and 20	FA by volume	58.86	7 and 13
Bideci et al. [24]	Length–25	5, 10 and 15	CA by volume	53.8	17, 20 and 47
	Length–50				52, 54 and 52
	Length–75				60, 61 and 58
AbdelAleem and Hassan [22]	<4.75	5, 10, 15 and 20	FA by volume	80.15	22, 39, 48 and 58
Ismail and Hassan [7]	0–4	5, 10, 15, 20, 25, 30 and 40	FA by volume	52.95	16, 21, 29, 42, 46, 53 and 67
Uygunoğlu and Topçu [23]	1–4	10, 20, 30, 40 and 50	FA by volume	approx. 25	12, 32, 42, 44 and 48
Yung et al. [38]	sieve #30 (0.6)	5, 10, 15 and 20	FA by volume	32.07	10, 22, 16 and 29
	sieve #50 (0.3)				4, 27, 27 and 32
	sieve #30 and #50				19, 25, 40 and 40
AbdelAleem et al. [28]	<4.75	5, 10, 15, 20, 25 and 30	FA by volume	75.7	12, 29, 41, 49, 51 and 58
Aslani et al. [5]	2	10, 20, 30 and 40	FA by volume	50.39	29, 41, 49 and 61
	5		FA by volume		19, 34, 41 and 48
	10		CA by volume		38, 56, 62 and 67
Najim and Hall [39]	2–6	5, 10 and 15	FA by weight	approx. 55	33, 42 and 53
			CA by weight		18, 40 and 58
			FA and CA by weight		18, 31 and 49
Emiroğlu et al. [6]	5–12	15, 30, 45 and 60	CA by weight	71.6	11, 34, 54 and 65
Güneyisi [25]	0–4	5, 15 and 25	FA by volume	73.1	21, 40 and 64
Topçu and Bilir [31]	0–4	8, 16.90 and 26.87	FA by weight	50.3	23, 40 and 71
Hilal [36]	0–1	5, 10, 15, 20 and 25	FA by volume	72.44	6, 12, 16, 21 and 31
	1–4				38, 18, 26, 37 and 46
	0–4				0, 10, 15, 21 and 32
Rahman et al. [1]	1–4	28	FA by volume	21.4	30
Turatsinze and Garros [26]	4–10	10, 15, 20 and 25	CA by volume	approx. 45	33, 54, 65 and 73
Ismail et al. [37]	<4.75	5, 10, 15, 20, 30 and 40	FA by volume	53.5	12, 19, 28, 39, 50 and 61
Bignozzi and Sandrolini [27]	0.5–2.0 and 0.05–0.7	22.2 and 33.3	FA by volume	33	25 and 39
Mishra and Panda [19]	5 and 10	5, 10, 15 and 20	CA by weight	65.4	11, 31, 39 and 47
Si et al. [14]	1.44–2.83	15 and 25	FA by volume	approx. 65	33 and 52
Khalil et al. [29]	0–2	10, 20, 30 and 40	FA by volume	approx. 27	17, 26, 37 and 40
Zaoiai et.al [21]	0/3	5	FA	37.9	37
	3/8	20	CA		36
Güneyisi et al. [35]	<1	5, 10, 15, 20 and 25	FA by volume	62.8	7, 18, 24, 31 and 39
	1–4				15, 21, 29, 42 and 50
	<4				13, 20, 27, 35 and 42
	10–40 length		CA by volume		17, 29, 36, 45 and 51
Aslani et al. [32]	5–10	20	CA by volume	50.39	56
Ismail and Hassan [33]	<4.75	5, 10, 15, 20, 25 and 30	FA by volume	75.65	12, 29, 41, 49, 51 and 58

**Table 4:** Influence of rubber size, replacement level and replaced material on 28-days flexural strength of SCC.

References	Rubber Size (mm)	Replacement Level (%)	Replaced Material	Initial Value (MPa)	Reduction (%)
AbdelAleem and Hassan [22]	<4.75	5, 10, 15 and 20	FA by volume	10.46	20, 35, 42 and 46
Ismail and Hassan [7]	0–4	5, 10, 15, 20, 25, 30 and 40	FA by volume	5.78	3, 9, 13, 20, 24, 31 and 42
Uygunoğlu and Topçu [23]	1–4	10, 20, 30, 40 and 50	FA by volume	approx. 1.7	6, 3, 9, 18 and 29
AbdelAleem et al. [28]	<4.75	0, 5, 10, 15, 20, 25 and 30	FA by volume	5.7	4, 11, 16, 19, 25 and 32
Najim and Hall [39]	2–6	5, 10 and 15	FA by weight	approx. 8.4	11, 15 and 35
			CA by weight		5, 27 and 39
			FA and CA by weight		10, 27 and 27
Hilal [36]	0–1	5, 10, 15, 20 and 25	FA by volume	5.6	12, 17, 20, 36 and 42
	1–4				15, 21, 25, 40 and 45
	0–4				14, 19, 23, 38 and 44
Ismail et al. [37]	<4.75	5, 10, 15, 20, 30 and 40	FA by volume	5.92	5, 7, 12, 16, 28 and 35
Mishra and Panda [19]	5 and 10	5, 10, 15 and 20	CA by weight	6.8	4, 9, 14 and 21
Khalil et al. [29]	0–2	10, 20, 30 and 40	FA by volume	approx. 3	17, 27, 30 and 33
Zaoiai et.al [21]	0/3	2.5 and 5	FA	7	4 and 24
	3/8	10 and 20	CA		4 and 37
Ismail and Hassan [33]	<4.75	5, 10, 15, 20, 25 and 30	FA by volume	5.74	5, 11, 16, 20, 26 and 32
Ganesan et al. [20]	0–4.75	15 and 20	FA by volume	5.65	15 and 10 (increase)

**Table 5:** Influence of rubber size, replacement level and replaced material on 28-days splitting tensile strength of SCC.

References	Rubber Size (mm)	Replacement Level (%)	Replaced Material	Initial Value (MPa)	Reduction (%)
AbdelAleem and Hassan [22]	< 4.75	5, 10, 15 and 20	FA by volume	4.73	17, 32, 34 and 41
Ismail and Hassan [7]	0–4	5, 10, 15, 20, 25, 30 and 40	FA by volume	4.19	1, 8, 20, 31, 38, 42 and 57
Aslani et al. [5]	2	10, 20, 30 and 40	FA by volume	3.7	8, 23, 27 and 39
	5		FA by volume		17 (increase), 15, 15 and 33
	10		CA by volume		21, 27, 32 and 50
AbdelAleem et al. [28]	<4.75	0, 5, 10, 15, 20, 25 and 30	FA by volume	4.5	9, 16, 20, 27, 33 and 40
Najim and Hall [39]	2–6	5, 10 and 15	FA by weight	approx. 4.5	29, 27 and 42
			CA by weight		18, 31 and 44
			FA and CA by weight		38, 29 and 47
Hilal [36]	0–1	5, 10, 15, 20 and 25	FA by volume	4.36	16, 19, 24, 28 and 44
	1–4				19, 23, 27, 36 and 49
	0–4				18, 20, 26, 34 and 48
Ismail et al. [37]	<4.75	5, 10, 15, 20, 30 and 40	FA by volume	4.35	2, 10, 23, 31, 42 and 52
Mishra and Panda [19]	5 and 10	5, 10, 15 and 20	CA by weight	4.65	5, 11, 19 and 24
Si et al. [14]	1.44–2.83	15 and 25	FA by volume	5.3	20 and 33
Khalil et al. [29]	0–2	10, 20, 30 and 40	FA by volume	approx. 3.4	4, 12, 9 and 26
Aslani et al. [32]	5–10	20	CA by volume	3.7	27
Ismail and Hassan [33]	<4.75	5, 10, 15, 20, 25 and 30	FA by volume	4.49	4, 13, 19, 24, 33 and 40

**Table 6:** Influence of rubber size, replacement level and replaced material on modulus of elasticity of SCC.

References	Rubber Size (mm)	Replacement Level (%)	Replaced Material	Initial Value (GPa)	Reduction (%)
Ismail and Hassan [7]	0–4	5, 10, 15, 20, 25, 30 and 40	FA by volume	33.61	6, 8, 18, 31, 32, 40 and 54
Uygunoğlu and Topçu [23]	1–4	10, 20, 30, 40 and 50	FA by volume	approx. 27	7, 15, 24, 35 and 44
AbdelAleem et al. [28]	<4.75	0, 5, 10, 15, 20, 25 and 30	FA by volume	29.4	6, 13, 16, 25, 32 and 36
Najim and Hall [39]	2–6	5, 10 and 15	FA by weight	approx. 43	2, 7 and 21
			CA by weight		12, 21 and 30
			FA and CA by weight		7, 16 and 26
Hilal [36]	0–1	5, 10, 15, 20 and 25	FA by volume	50.71	4, 9, 12, 21 and 33
	1–4				10, 18, 26, 29 and 39
	0–4				8, 15, 17, 25 and 35
Rahman et al. [1]	1–4	28	FA by volume	approx. 24.5	10 to 20
Turatsinze and Garros [26]	4–10	10, 15, 20 and 25	CA by volume	approx. 35	34, 46, 57 and 71
Ismail et al. [37]	<4.75	5, 10, 15, 20, 30 and 40	FA by volume	33.59	4, 8, 19, 28, 34 and 46
Bignozzi and Sandrolini [27]	0.5–2.0 and 0.05–0.7	22.2 and 33.3	FA by volume	33	19 and 28
Si et al. [14]	1.44–2.83	15 and 25	FA by volume	4308 m/s	6 and 7
Ismail and Hassan [33]	<4.75	5, 10, 15, 20, 25 and 30	FA by volume	29.37	6, 12, 16, 25, 32 and 36

**Table 7:** Influence of rubber inclusion on ductility of self-compacting rubberized concrete (SCRC) and rubberized concrete (RC).

References	Concrete Type	Rubber Type	Rubber Size (mm)	Replacement Level (%)	Replaced Material	Ductility
Hilal [36]	SCRC	crumb rubber	0–4	5, 10, 15, 20, 25	FA	increased
Vadivel et al. [41]	RC	crumb rubber tire chips	0–2.36 and 20	3, 6	FA and CA	increased
Gesoğlu et al. [8]	RC	crumb rubber	1–4	5, 10 and 20	FA	increase
		tire chips	< 10	5, 10 and 20	CA	decreased
		fine crumb rubber	0.1–1.00	5, 10 and 20	FA	decreased
Youssf et al. [42]	RC	crumb rubber	1.18 and 2.36 and 0.15–2.36	0 to 20	FA	increased
Zheng et al. [43]	RC	rubber powder or rubber chips	<2.6 and 4–1	15, 30 and 45	CA	increased
Elghazouli et al. [12]	RC	tyre rubber	0–2	45 and 60	FA and CA	increased
Gesoğlu et al. [44]	RC	crumb rubber and tire chips	<4 and 10–40 length	5, 10, 15, 20, 25, 30	FA and CA	increased

**Table 8:** Rubber size and replaced material (fine and/or coarse aggregate) of different authors.

References	Rubber Size	Replaced Material
AbdelAleem and Hassan [22]	<4.75 mm	FA
AbdelAleem et al. [28]	<4.75 mm	FA
Aslani et al. [5]	2 mm, 5 mm, 10 mm	FA and CA
Aslani et al. [32]	5–10 mm	CA
Bideci et al. [24]	75, 50 and 25 mm length; 5 × 5 mm cross section	CA
Bignozzi and Sandrolini [27]	0.5–2.0 mm, 0.05–0.7 mm	FA
Emiroğlu et al. [6]	5–12 mm	CA
Ganesan et al. [20]	<4.75 mm	FA
Gesoğlu and Güneyisi [46]	<4 mm	FA
Güneyisi [25]	<4 mm	FA
Güneyisi et al. [35]	0–1 mm (No. 18), 1–4 mm (No. 5), 0–4 mm (No.18 and No. 5) and 10 to 40 mm length	FA and CA
Hilal [36]	0–4 mm	FA
Ismail et al. [37]	<4.75 mm	FA
Ismail and Hassan [7]	0–4 mm	FA
Ismail and Hassan [33]	<4.75 mm	FA
Khalil et al. [29]	0–2 mm	FA
Jedidi et al. [15]	size 0/4 and 4/8	CA
Mishra and Panda [19]	5 mm and 10 mm	CA
Najim and Hall [39]	2–6 mm	FA and CA
Najim and Hall [34]	2–6 mm	FA and CA
Rahman et al. [1]	1–4 mm	FA
Si et al. [14]	1.44–2.83 mm	FA
Topçu and Bilir [31]	0–4 mm	FA
Turatsinze and Garros [26]	4–10 mm	CA
Uygunoğlu and Topçu [23]	1–4 mm	FA
Yung et al. [38]	sieve #30 and #50	FA
Zaoiai et al. [21]	0/3 or 3/8	FA and CA
